# Fluid-Attenuated Inversion Recovery Vascular Hyperintensity as a Potential Predictor for the Prognosis of Acute Stroke Patients After Intravenous Thrombolysis

**DOI:** 10.3389/fnins.2021.808436

**Published:** 2022-01-25

**Authors:** Lin Zhu, Fuping Jiang, Meng Wang, Qian Zhai, Qing Zhang, Feng Wang, Xuqiang Mao, Nihong Chen, Junshan Zhou, Guangjun Xi, Yachen Shi

**Affiliations:** ^1^Department of Neurology, Nanjing First Hospital, Nanjing Medical University, Nanjing, China; ^2^Department of Neurology, The Affiliated Wuxi People’s Hospital of Nanjing Medical University, Wuxi, China

**Keywords:** acute stroke, intravenous thrombolysis, fluid-attenuated inversion recovery vascular hyperintensity, prognosis, survival

## Abstract

**Background:**

Fluid-attenuated inversion recovery vascular hyperintensity (FVH) can reflect the collateral status, which may be a valuable indicator to predict the functional outcome of acute stroke (AS) patients.

**Methods:**

A total of 190 AS patients with large vessel occlusion (LVO) were retrospectively investigated. All patients completed a 6-month follow-up and their modified Rankin Scale (mRS) scores were recorded at 1, 3, and 6 months after intravenous thrombolysis (IVT). Based on their mRS at 3 months, patients were divided into two groups: poor prognosis (131 patients; 68.9% of all subjects) and favorable prognosis (59 patients; 31.1% of all subjects). The death records of 28 patients were also analyzed in the poor prognosis group.

**Results:**

(1) Univariate and multivariate analyses showed that the higher National Institutes of Health Stroke Scale (NIHSS) score at admission, higher fasting blood glucose, and lower FVH score were independent risk factors to predict the poor prognosis of IVT. (2) Survival analysis indicated that FVH score was the only baseline factor to predict the 6-month survival after IVT. (3) Baseline FVH score had great prediction performance for the prognosis of IVT (area under the curve = 0.853). (4) Baseline FVH score were negatively correlated with the NIHSS score at discharge and mRS score at 1, 3, and 6 months.

**Conclusion:**

Among various baseline clinical factors, only the FVH score might have implications for 3-month outcome and 6-month survival of AS patients after IVT. Baseline FVH score showed great potential to predict the prognosis of the AS patients.

## Introduction

As shown by epidemiology analyses, stroke is the main cause of death in China, with the largest number of stroke cases worldwide, thus increasing the medical and nursing burden in this country (Wu et al., 2019; Roth and Mohamadzadeh, 2021). Currently, intravenous thrombolysis (IVT), which is used as the first-line treatment of acute stroke (AS) within 4.5 h of symptom onset, has demonstrated benefits for reducing mortality (Wardlaw et al., 2014; Rabinstein, 2020). However, in clinical practice, the efficacy of IVT may be affected by many factors, such as blood pressure (Wang et al., 2020), fasting blood glucose (Zhang et al., 2019), C-reactive protein (CRP) (Karlinski et al., 2014), among others. Furthermore, the baseline collaterals status may have an important effect on the prognosis and functional recovery after IVT in AS patients (Bang et al., 2015; Singer et al., 2015; Ravindran et al., 2021). Hence, it is essential to investigate factors that can potentially predict the prognosis after IVT to better identify patients who are likely to respond to IVT as well as implement early more aggressive therapies, such as endovascular intervention, for those patients with poor prognosis.

Fluid-attenuated inversion recovery vascular hyperintensity (FVH), firstly reported by Cosnard et al. (1999), indicates slow retrograde flow in leptomeningeal collaterals and is related to abnormal hemodynamics of the brain (Azizyan et al., 2011; Ding et al., 2020). However, controversy surrounds the underlying mechanisms and clinical implications of FVH. Certain authors have showed that FVH is associated with poor collaterals and worst clinical outcome (Kufner et al., 2015; Nave et al., 2018), while other studies associated FVH with good collaterals and favorable prognosis (Olindo et al., 2012; Mahdjoub et al., 2018). Differences in populations or FVH classifications may be the main culprit of these inconsistent results (Li et al., 2021; Wang et al., 2021). Our previous study demonstrated that a higher FVH score based on the Alberta Stroke Program Early CT Score (ASPECTS) grading system was associated with better collaterals and functional outcome after endovascular therapy in AS patients with large vessel occlusion (LVO) (Jiang et al., 2019). Therefore, FVH score may be a valuable predictor of functional outcome. However, the potential clinical value of FVH score after IVT in AS patients remains unclear, especially for the prediction of survival outcome of IVT.

In the present study, we aimed to investigate the baseline risk factors for influencing the therapeutic effect of IVT in AS patients and to assess the predictive value of these factors for the clinical outcome of IVT. We hypothesized that FVH score reflecting the collateral status can predict the prognosis of AS patients after IVT.

## Materials and Methods

### Participants

The present study is a retrospective study. A total of 190 AS patients who received IVT according to a stroke registry of Nanjing First Hospital (Gong et al., 2019; Jiang et al., 2019) were included in the present study. The protocol was approved by the ethics committee of Nanjing First Hospital, Nanjing Medical University. All patients or their legal guardians provided informed consent.

The inclusion criteria were as follows: patients (I) presenting a first-ever AS with LVO or a previous stroke with hemiplegia sequelae that did not affect the neurological score; (II) conducting pre-treatment magnetic resonance imaging (MRI) scans including diffusion weighted imaging (DWI) and fluid-attenuated inversion recovery (FLAIR); (III) accepting IVT within 4.5 h after symptom onset; and (IV) completing a 6-month follow-up with the complete records of the modified Rankin Scale (mRS) assessment. Additionally, the exclusion criteria were: (I) age < 18 years old; (II) severe comorbidities, e.g., renal failure, hepatic failure, systemic inflammatory disease, cancer, and presence of cerebral hemorrhage or trauma; (III) any contraindication of MRI; (IV) refusal of thrombolysis or hospital transfer during therapy; and (V) inability to analyze MRI due to low-quality imaging.

### Acquisition of Medical Data

Clinical data of each patient were extracted from the Nanjing First Hospital Stroke registry database (Chen et al., 2019). The information collected consisted of age, sex, and vascular risk factors (i.e., hypertension, diabetes mellitus, hyperlipidemia, coronary heart disease, atrial fibrillation, hemorrhagic transformation, previous stroke, current drinking, and current smoking). Furthermore, patients were assessed using the National Institutes of Health Stroke Scale (NIHSS) at admission and discharge, and the mRS value was also determined at 1, 3, and 6 months. In addition, other clinical parameters were obtained at baseline, including levels of door-to-needle time, blood pressure, platelet count, serum creatinine, fasting blood glucose, triglyceride, low-density lipoprotein cholesterol, high-density lipoprotein cholesterol, homocysteine, high-sensitivity CRP (hs-CRP), and hemoglobin A_1c_.

### Imaging Acquisition and Analysis

A 3.0 T MRI scanner (Ingenia, Philips Medical Systems) was used to conduct the MRI scans, according to the protocol in our previous study (Jiang et al., 2019). Briefly, the various parameters employed were as follows: (I) DWI sequence: relaxation time/echo time (TR/TE) = 2,501/98 ms; *b*-values = 0–1,000 s/mm^2^; matrix size = 152 × 122; slice thickness = 6 mm; flip angle (FA) = 90°; field of view (FOV) = 230 × 230 mm; slices = 18; section thickness = 6 mm; and intersection gap = 1.3 mm and (II) FLAIR sequence: TR/TE = 7,000/120 ms; matrix size = 356 × 151; slice thickness = 6 mm; FA = 90°; FOV = 230 × 230 mm; slices = 18; section thickness = 6 mm; and intersection gap = 1.3 mm.

Based on the spatial distribution of FVHs in the ASPECTS cortical areas (insula, M1-M6) and on data from our previous study (Jiang et al., 2019), two professional neurologists blinded to the clinical data provided the FVH score for each patient. The FVH score ranged from 0 to 7—if the image showed no FVH, the FVH score was 0, whereas if FVHs abutted all ASPECTS cortical areas, the FVH score was 7. As the two researchers completed the analysis of each image independently, any inconsistent FVH score proposed between them would be reviewed for a consensus.

### Statistical Analysis

All statistical analyses were performed using SPSS 16.0 software (SPSS, Inc., Chicago, IL) or GraphPad Prism 8.0 (GraphPad Software Inc., United States). Continuous data is presented as the mean ± standard deviation if variables followed a normal distribution, while variables of non-normal distribution are presented as medians [interquartile range (IQR)]. A chi-squared test was used for the categorical variables and an independent-sample *t*-test or Mann-Whitney *U*-test was used for univariate analyses of continuous variables. Multivariate analysis was conducted using the logistic regression model to identify potential predictors, and the odds radio and 95% confidence interval (CI) were obtained. Furthermore, to evaluate the prognostic significance of potential predictors for the 6-month survival of patients, Kaplan-Meier curves were plotted and the Cox regression analysis was conducted. The receiver operating characteristic (ROC) curve analysis was performed to assess the predictive power of potential prognosis predictors. Spearman correlation analysis was used to find associations between potential predictors and prognosis assessment. Results were considered statistically significant if *P*-values were < 0.05.

## Results

### Characteristics of the Study Cohort

According to the assessment of the mRS at 3 months, 190 patients with AS were classified into two groups: 131 patients with poor prognosis (68.9%) and 59 patients with favorable prognosis (31.1%). The comparison of clinical characteristics between the two groups using the univariate analysis is shown in Table 1. At baseline, the poor prognosis group showed significantly higher NIHSS score at admission, systolic blood pressure, fasting blood glucose, serum creatinine, and serum hs-CRP, and significantly lower FVH score compared with the favorable prognosis group (all *P* < 0.05). Additionally, after IVT, the NIHSS score at discharge, as well as the mRS at 1, 3, and 6 months, were worse in patients with poor prognosis than those with favorable prognosis (*P* < 0.001). However, there was no significant difference in other characteristics between the two groups (all *P* > 0.05).

**TABLE 1:** Comparison of characteristics between patients with poor and favorable prognosis after intravenous thrombolysis.

Variables	Poor prognosis (*n* = 131)	Favorable prognosis (*n* = 59)	*P*-value
Age (years)	74 (66–80)	69 (62–79)	0.126^c^
Sex, male	80 (61.07%)	42 (71.19%)	0.178^b^
Hypertension	95 (72.52%)	38 (61.02%)	0.259^b^
Diabetes mellitus	34 (25.95%)	9 (15.25%)	0.103^b^
Hyperlipidemia	6 (4.58%)	5 (8.47%)	0.288^b^
Coronary heart disease	35 (26.72%)	16 (27.12%)	0.954^b^
Atrial fibrillation	29 (22.14%)	11 (18.64%)	0.585^b^
Previous stroke	33 (25.19%)	13 (22.03%)	0.638^b^
Hemorrhagic transformation	24 (18.32%)	9 (15.25%)	0.606^b^
Alcohol drinking	35 (26.72%)	22 (37.29%)	0.324^b^
Smoking	49 (37.40%)	30 (50.85%)	0.082^b^
NIHSS at admission	13.47 ± 6.55	9.24 ± 4.86	<0.001^a^
DNT (min)	38 (25–60)	39 (25–60)	0.667^c^
Baseline detection			
SBP (mmHg)	147.45 ± 23.17	137.25 ± 20.86	0.004^a^
DBP (mmHg)	88.52 ± 16.20	84.59 ± 13.60	0.107^a^
PC ( × 10^9/L)	180.50 ± 65.05	194.34 ± 63.83	0.174^a^
Creatinine (μmol/L)	72 (59–93)	68 (57–78)	0.039^c^
FBG (mmol/L)	6.36 (5.28–7.86)	5.40 (4.62–6.91)	0.002^c^
TC (mmol/L)	4.43 (3.62–5.31)	4.16 (3.40–5.10)	0.233^c^
TG (mmol/L)	1.10 (0.81–1.42)	1.04 (0.65–1.59)	0.623^c^
LDL-c (mmol/L)	2.75 ± 0.90	2.56 ± 1.05	0.184^a^
HDL-c (mmol/L)	1.19 (0.95–1.37)	1.17 (0.93–1.29)	0.470^c^
Homocysteine (μmol/L)	7.86 (2.65–12.50)	6.75 (3.63–10.74)	0.710^c^
hs-CRP (μg/mL)	13.59 (10.44–17.25)	12.08 (9.52–15.00)	0.045^c^
HbA_1c_, %	6.00 (5.50–6.90)	5.80 (5.50–6.50)	0.411^c^
FVH score	1 (0–3)	4 (4–5)	<0.001^c^
NIHSS at discharge	11 (7–17)	2 (1–4)	<0.001^c^
mRS at 1 month	4 (4–5)	1 (1–3)	<0.001^c^
mRS at 3 months	4 (3–5)	1 (0–2)	<0.001^c^
mRS at 6 months	3 (3–5)	1 (0–2)	<0.001^c^

*Data is presented as mean ± standard deviation / number (percentage of total)/median (25 percentile–75 percentile).*

*NIHSS, National Institutes of Health Stroke Scale; DNT, door-to-needle time; SBP, systolic blood pressure; DBP, diastolic blood pressure; PC, platelet count; FBG, fasting blood glucose; TC, total cholesterol; TG, triglyceride; LDL-c, low-density lipoprotein cholesterol; HDL-c, high- density lipoprotein cholesterol; hs-CRP, high-sensitivity C-reactive protein; HbA_1c_, hemoglobin A_1c_; FVH, Fluid-attenuated inversion recovery vascular hyperintensity; mRS, modified Rankin scale.*

*^a^Independent-sample t-test.*

*^b^Chi-squared test.*

*^c^Mann-Whitney U-test.*

To comprehensively assess the association between baseline features and patient prognosis (poor / favorable), we performed a multivariate analysis followed by logistics analysis. As displayed subsequently, and the results were displayed in Table 2. The logistics analysis revealed that NIHSS score at admission (OR = 1.214, 95% CI = 1.084 ∼ 1.361, *P* = 0.001), fasting blood glucose (OR = 1.385, 95% CI = 1.004 ∼ 1.911, *P* = 0.045), and FVH score (OR = 0.349, 95% CI = 0.245 ∼ 0.497, *P* < 0.001) were related to poor prognosis onset, and might serve as independent risk factors to predict poor prognosis in AS patients receiving IVT.

**TABLE 2:** Logistics analysis for the association between baseline clinical features and prognosis in all patients.

Variables	OR	95%CI	*P*-value
Age	0.965	0.906–1.028	0.265
Sex	0.605	0.152–2.411	0.476
Hypertension	1.586	0.475–5.294	0.453
Diabetes mellitus	2.290	0.484–10.838	0.296
Hyperlipidemia	0.432	0.065–2.865	0.385
Coronary heart disease	1.417	0.374–5.366	0.608
Atrial fibrillation	1.031	0.230–4.620	0.969
Previous stroke	0.637	0.141–2.879	0.558
Hemorrhagic transformation	0.751	0.165–3.430	0.712
Alcohol drinking	0.964	0.237–3.918	0.959
Smoking	0.756	0.147–3.880	0.737
NIHSS at admission	1.214	1.084–1.361	0.001
DNT	0.998	0.982–1.014	0.783
SBP	1.032	0.996–1.069	0.080
DBP	0.971	0.923–1.021	0.248
PC	0.995	0.987–1.004	0.272
Creatinine	1.032	0.998–1.067	0.068
FBG	1.385	1.004–1.911	0.045
TC	0.958	0.842–1.090	0.517
TG	0.976	0.446–2.134	0.951
LDL-c	1.459	0.776–2.745	0.241
HDL-c	1.239	0.347–4.426	0.741
Homocysteine	1.002	0.949–1.059	0.929
hs-CRP	1.105	0.969–1.260	0.136
HbA_1c_	0.990	0.882–1.111	0.861
FVH score	0.349	0.245–0.497	<0.001

*CI, confidence interval; NIHSS, National Institutes of Health Stroke Scale; DNT, door-to-needle time; SBP, systolic blood pressure; DBP, diastolic blood pressure; PC, platelet count; FBG, fasting blood glucose; TC, total cholesterol; TG, triglyceride; LDL-c, low-density lipoprotein cholesterol; HDL-c, high-density lipoprotein cholesterol; hs-CRP, high-sensitivity C-reactive protein; FVH, Fluid-attenuated inversion recovery vascular hyperintensity.*

### Prognostic Value for the Survival Outcomes of Patients With Poor Prognosis

**FIGURE 1:**
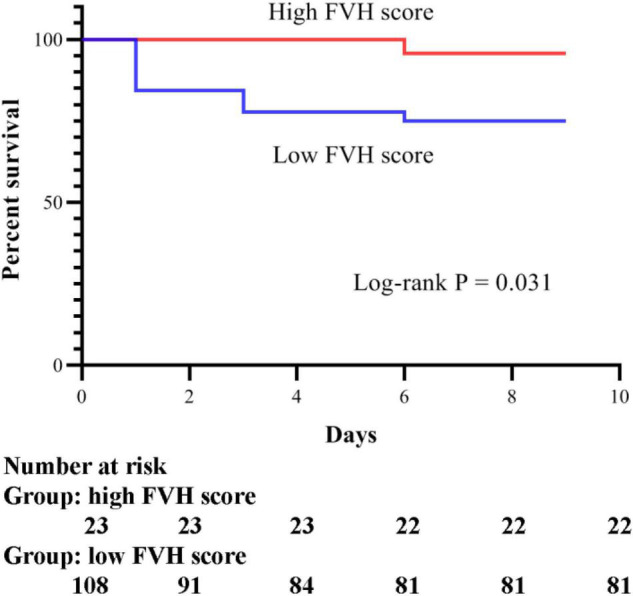
Kaplan-Meier survival curves for acute stroke patients.

**FIGURE 2:**
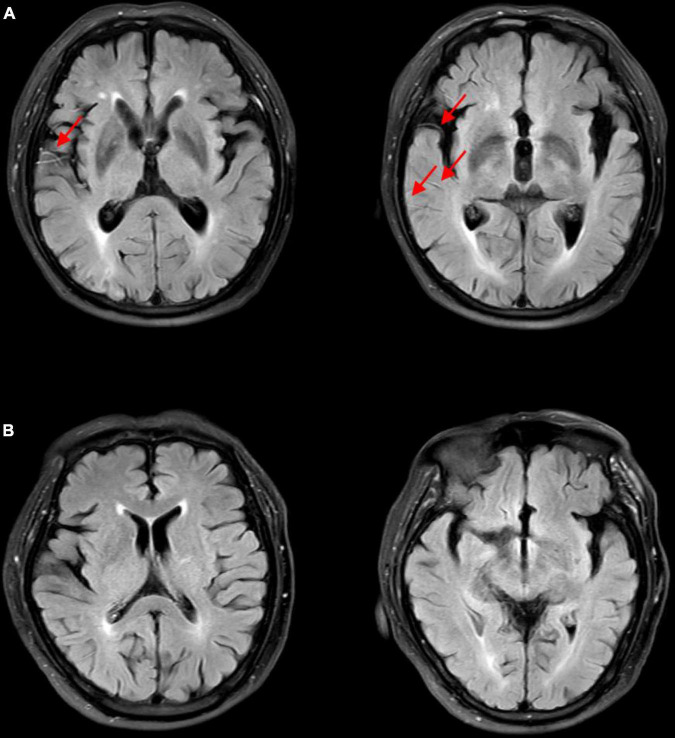
FLAIR images of representative “high” and “low” FVH scores in acute stroke patients. **(A)** High FVH scores (FVH = 6). Red arrows show FVHs. **(B)** Low FVH scores (FVH = 0). FVH, fluid-attenuated inversion recovery vascular hyperintensity.

According to the 6-month follow-up records, 28 patients died. Therefore, we evaluated the survival of patients with poor prognosis in the present study. The Cox regression model was used to assess baseline risk factors to predict the survival outcomes. As shown in Table 3, our results indicated that only the FVH score could independently predict the survival of patients and can thus be used as a potential prognostic biomarker for IVT (*P* = 0.009). In addition, we analyzed the Kaplan-Meier survival curves (illustrated in Figure 1). To facilitate the survival analysis, FVH scores were divided into low (0–3 score) and high (4–7 score) (Jiang et al., 2019; Li et al., 2021), indicating that patients with lower FVH score had a significantly lower 6-month survival than those with higher FVH score (log-rank *P* = 0.031). Figure 2 shows two images of representative “high” and “low” FVH scores in AS patients.

**TABLE 3:** Cox regression analysis in patients with poor prognosis.

Variables	HR	95%CI	*P*-value
Age	0.992	0.945–1.043	0.760
Sex	1.021	0.366–2.854	0.968
Hypertension	1.445	0.463–4.511	0.526
Diabetes mellitus	0.700	0.197–2.482	0.581
Hyperlipidemia	0.585	0.045–7.620	0.682
Coronary heart disease	2.271	0.704–7.322	0.170
Atrial fibrillation	0.805	0.259–2.501	0.708
Previous stroke	1.241	0.38 –4.011	0.718
Hemorrhagic transformation	2.138	0.678–6.746	0.195
Alcohol drinking	1.265	0.336–4.761	0.729
Smoking	0.869	0.231–3.261	0.835
NIHSS at admission	1.042	0.968–1.121	0.274
DNT	0.985	0.967–1.003	0.109
SBP	0.990	0.964–1.016	0.436
DBP	1.016	0.983–1.051	0.342
PC	0.996	0.989–1.004	0.996
Creatinine	1.005	1.000–1.011	0.060
FBG	1.210	0.987–1.482	0.067
TC	1.921	0.488–7.568	0.351
TG	0.478	0.160–1.424	0.185
LDL-c	0.758	0.162–3.536	0.724
HDL-c	0.972	0.505–1.869	0.931
Homocysteine	1.029	0.997–1.063	0.073
hs-CRP	0.997	0.983–1.011	0.675
HbA_1c_	0.966	0.845–1.105	0.615
FVH score	0.584	0.391–0.872	0.009

*CI, confidence interval; NIHSS, National Institutes of Health Stroke Scale; DNT, door-to-needle time; SBP, systolic blood pressure; DBP, diastolic blood pressure; PC, platelet count; FBG, fasting blood glucose; TC, total cholesterol; TG, triglyceride; LDL-c, low-density lipoprotein cholesterol; HDL-c, high-density lipoprotein cholesterol; hs-CRP, high-sensitivity C-reactive protein; FVH, Fluid-attenuated inversion recovery vascular hyperintensity.*

### Predictive Performance of Baseline Fluid-Attenuated Inversion Recovery Vascular Hyperintensity Score for the Poor/Favorable Prognosis in Patients After Intravenous Thrombolysis

**FIGURE 3:**
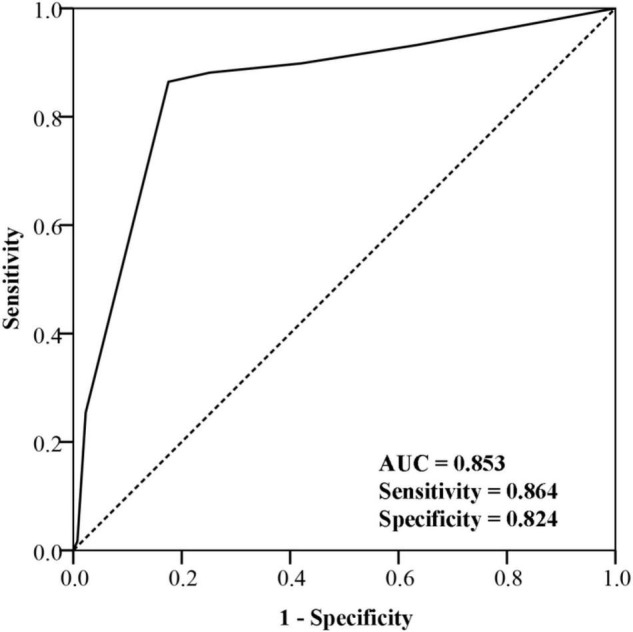
Receiver operating characteristic curve of fluid-attenuated inversion recovery vascular hyperintensity score for prognosis prediction.

ROC curves shown in Figure 3 suggested that baseline FVH score could help distinguish patients with poor prognosis from those with favorable prognosis accurately. The area under the curve (AUC) was 0.853, while the sensitivity and specificity were 0.864 and 0.824, respectively.

### Correlation of Baseline Fluid-Attenuated Inversion Recovery Vascular Hyperintensity Score With the Assessments of Prognosis in Patients After Intravenous Thrombolysis

**FIGURE 4:**
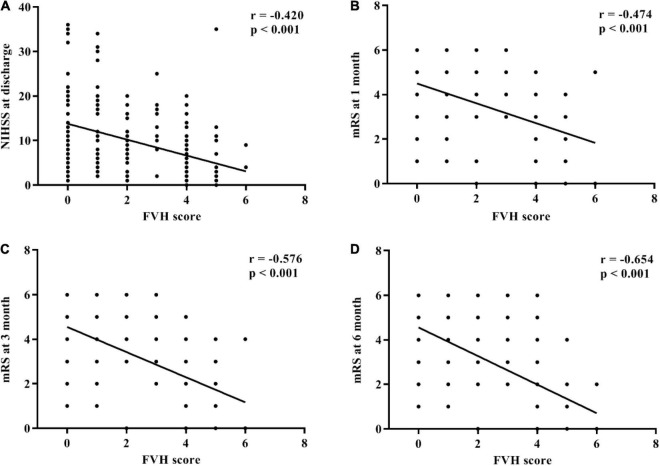
Correlation between fluid-attenuated inversion recovery vascular hyperintensity score and symptom assessments after intravenous thrombolysis. **(A)** National Institutes of Health Stroke Scale (NIHSS) at admission. **(B)** Modified Rankin Scale at 1 month. **(C)** Modified Rankin Scale at 3 months. **(D)** Modified Rankin Scale at 6 months.

Spearman’s rank correlation analysis revealed that FVH score negatively correlated with the following parameters: NIHSS score at discharge (*r* = −0.420, *p* < 0.001; Figure 4A), mRS at 1 month (*r* = −0.474, *p* < 0.001; Figure 4B), mRS at 3 months (*r* = −0.576, *p* < 0.001; Figure 4C), and mRS at 6 months (*r* = −0.654, *p* < 0.001; Figure 4D).

## Discussion

The key findings in the present study were as follows: (I) NIHSS score at admission, systolic blood pressure, fasting blood glucose, serum creatinine, serum hs-CRP, and FVH score were significantly different between patients with poor and favorable prognosis; (II) multivariate analysis indicated that NIHSS score at admission, fasting blood glucose, and FVH score might be independent risk factors to predict prognosis of IVT; (III) FVH score was the most important baseline factor to predict the 6-month survival after IVT; (IV) baseline FVH score showed a high prediction performance for the outcome of IVT (AUC = 0.853) and significantly negative correlation with NIHSS score at discharge and mRS score at 1, 3, and 6 months. Taken together, compared with various risk factors at baseline, baseline FVH score is a promising biomarker to predict the clinical outcome of IVT in AS patients, especially to predict survival after IVT.

The present work is a retrospective study to investigate the potential baseline factors affecting the prognosis of IVT in AS patients. The main strengths of our study were the large sample size with complete clinical and imaging data, and completing the longest follow-up period to our knowledge for assessing adequately the predicting potential of AS patient survival after IVT. Additionally, a progressive data analysis (univariate analysis—multivariate analysis—survival analysis) was performed to strictly identify the optimal baseline predictor. Furthermore, to decrease the heterogeneity of cohorts, we only analyzed the clinical data of AS patients with LVO. Hence, the present findings are based on solid evidence and have important clinical value to guide the treatment of AS stroke.

In the present study, we found that NIHSS score at admission, systolic blood pressure, fasting blood glucose, serum creatinine, and serum hs-CRP were baseline risk factors influencing the prognosis of AS patients after IVT. Mehta et al. (2017) reported that an NIHSS score at admission lower than 15 was indicative of a milder baseline stroke severity in AS patients, who could benefit from IVT for a favorable outcome. Similar findings were also described in other studies (Sobolewski et al., 2013; Kim et al., 2018; Xiong et al., 2021), suggesting that lower NIHSS scores at admission may be an important clinical factor associated with favorable outcome after IVT. In addition, higher systolic blood pressure and fasting blood glucose levels can increase the burden of cerebral blood vessels and exacerbate damage to blood vessels (Mayhan et al., 1987; Cao et al., 2015). Since blood pressure and fasting glucose are two key references of metabolic syndrome (Bas and Ozdemir, 2017), these factors may be related to the poor prognosis of AS patients treated with IVT. Furthermore, previous studies have demonstrated that elevated serum creatinine levels within the normal range predict an increased risk of cerebrovascular disease and are therefore considered an independent predictor of survival after stroke (Friedman, 1991; Wannamethee et al., 1997). Hence, relatively higher serum creatinine concentration, reflecting a slight impairment of renal function, may be related to the pathogenesis of stroke and affect IVT efficacy. Additionally, stroke-associated infections have been demonstrated to affect clinical outcome in AS stroke using IVT (Derbisz et al., 2021), while higher CRP levels have been associated with functional outcome and cognitive outcome at discharge (Irimie et al., 2018), which suggests that high serum CRP may predict the future vascular events and poor prognosis. Consequently, the present findings highlighted the importance of these risk factors at baseline for prognosis prediction, leading to an individualized therapy strategy that may contribute to achieving a favorable clinical outcome.

This is the first study to propose that, compared with other baseline factors, baseline FVH score not only affected long-term outcome but also mortality. As a common observation in stroke, FVH reflects the damaged hemodynamics and retrograde collateral blood flow characteristic of large arterial occlusions, for which it is considered a valuable arterial occlusion marker (Azizyan et al., 2011; Cheng et al., 2012; Ding et al., 2020; Lee et al., 2021). Recent studies have detected that the distribution of collateral vessels could be reflected by FVH score during the arterial occlusion and that the greater FVH score before therapy represented, the greater the collateral status (Mahdjoub et al., 2018; Nave et al., 2018; Jiang et al., 2019). Hence, baseline FVH may be a vital indicator in relation to the clinical benefits of recanalization. Based on previous reports (Lee et al., 2009; Jiang et al., 2019; Nomura et al., 2020), the present study demonstrated that higher baseline FVH scores represented a good collateral status and a favorable prognosis at 3 months in AS patients after IVT. Most importantly, we also found that baseline FVH scores < 4 exhibited a low survival rate within 6 months after IVT, suggesting that the insufficient collateralization before therapy may elevate long-term risk of death and that baseline FVH score may be a potential risk indicator to predict patient survival. Simultaneously, ROC analysis further demonstrated an excellent prediction performance of baseline FVH score for the prognosis after IVT. In addition, our correlation analysis indicated that baseline FVH score can reflect the severity of stroke symptoms in multiple stages after therapy, further supporting baseline FVH score as a stable predictor for the therapeutic effect of IVT. Altogether, the assessment of FVH before therapy was useful and essential for predicting functional outcome and survival.

There were some limitations to this study. (I) Despite the LVO, many AS patients show the non-LVO, e.g., cardioembolic stroke, cerebral small vessel disease. The potential relationship between baseline risk factors and non-LVO are not explored in the present study. In subsequent research, we will investigate populations with other stroke types to assess their baseline risk factors for the prognosis after IVT. (II) Time of survival is expressed in “months” for survival analysis because the exact day records are incomplete. Additionally, if AS patients had a survival time over 6 months, we assigned them as “survival time = 9” for drawing the survival curve. In the future study, we will further extend the follow-up period (e.g., 12 months) to provide more significant information, such as detailed survival time, assessments of daily activities, cognition, and sleep.

## Conclusion

In conclusion, NIHSS score at admission, systolic blood pressure, fasting blood glucose, serum creatinine, serum hs-CRP, and FVH score may be baseline risk factors that affect the efficacy of IVT in clinic. Among them, only FVH score may be implicated in the 3-month outcome and 6-month survival of AS patients after IVT. Therefore, baseline FVH score could be a promising clinical predictor for prognosis.

## Data Availability Statement

The original contributions presented in the study are included in the article/supplementary material, further inquiries can be directed to the corresponding author/s.

## Ethics Statement

The studies involving human participants were reviewed and approved by the Ethics Committee of Nanjing First Hospital, Nanjing Medical University. The patients/participants provided their written informed consent to participate in this study.

## Author Contributions

LZ drafted the manuscript and contributed to the discussion. FJ collected the data and analyzed the data. NC analyzed the data. FW and XM contributed to the discussion. GX contributed to the discussion and revised the manuscript. MW, QiaZ, and QinZ contributed to collecting and recording data of the stroke database. JZ contributed to the quality control of the stroke registry database. YS designed the study and revised the manuscript. All authors contributed to the article and approved the submitted version.

## Conflict of Interest

The authors declare that the research was conducted in the absence of any commercial or financial relationships that could be construed as a potential conflict of interest.

## Publisher’s Note

All claims expressed in this article are solely those of the authors and do not necessarily represent those of their affiliated organizations, or those of the publisher, the editors and the reviewers. Any product that may be evaluated in this article, or claim that may be made by its manufacturer, is not guaranteed or endorsed by the publisher.
